# Synthesis and Characterization of Biodegradable Semi-Interpenetrating Polymer Networks Based on Star-Shaped Copolymers of ɛ-Caprolactone and Lactide

**Published:** 2017

**Authors:** Ali Hossein Rezayan, Negar Firoozi, Somayyeh Kheirjou, Seyed Jamal Tabatabaei Rezaei, Mohammad Reza Nabid

**Affiliations:** a *Department of Life Science Engineering, Faculty of New Sciences and Technologies, University of Tehran, P.O. Box 14395-1561, Fax +982188617087,Tehran, Iran. *; b *Department of Chemistry, Faculty of Science, University of Zanjan, P.O. Box 45195-313, Zanjan, Iran.*; c *Faculty of Chemistry, Shahid Beheshti University, G.C., P.O. Box 1983963113, Tehran, Iran.*

**Keywords:** polymer network, polyurethane, biodegradable, biocompatible, soft tissue engineering

## Abstract

In this paper, the focus is on a new kind of biodegradable semi-interpenetrating polymer networks, which is derived from ɛ-caprolactone, lactide, 1,4-butane diisocyanate and ethylenediamine and also its potential has been investigated in soft tissue engineering applications. The polymers were characterized by nuclear magnetic resonance (NMR) spectrometry, Fourier transform infrared spectroscopy (FT-IR), differential thermal analysis (DTA), differential scanning calorimetry (DSC), and thermogravimetric analysis (TGA). These experiments show that the polymers with the right composition and the expected molecular weight were achieved. Also, the *in-vi*tro degradation of polymer network was examined in phosphate buffer solutions (pH 7.4) at 37 °C. Moreover, cell viability and adhesion tests were carried out with fibroblast cells by the MTT assay, which confirmed biocompatibility. Polyurethane materials have superior mechanical properties, so these biodegradable and biocompatible films demonstrate potential for future application as cell scaffolds in soft tissue engineering applications.

## Introduction

Biodegradable random and block copolymers are an attractive class of polymeric materials which belong to a big family known as “soft materials”. The biodegradability, biocompatibility, and permeability of polyesters such as polylactide (PLA), poly(ε-caprolactone) (PCL), polyglycolide (PGA), and their copolymers bring them to a considerable interest in many drugs and clinical demands from tissue engineering to medical devices (1-4). PLA and PCL have been successfully used for the synthesis of numerous star, multi-arm star, and also hyperbranched polymers (5, 6).

Star-shaped macromolecules were widely used as model-branched polymers for evaluating the effect of branching on the polymers properties. These polymeric species consist of a central core and some linear chains linked to that core by one of their ends. Various paths for the preparation of star-shaped polymers have been described in the literature. The study of physical properties of branched polymers is one of the recent topics in polymer science and this is not only caused by the practical importance of these types of polymers but it is also related to their importance in examining the basic theories in polymer science (7-11).

Semi-interpenetrating polymeric networks (semi-IPNs) have appeared as new biomaterials as scaffolds for tissue engineering. The IUPAC definition of IPN is: “a polymer comprising one or more networks and one or more linear or branched polymer(s) characterized by the penetration on a molecular scale of at least one of the networks by at least some of the linear or branched macromolecules” (12, 13). Comparing to individual cross-linked networks, physical entanglements, and network interactions cause semi-IPN help improve the mechanical strength and resiliency of the polymer. Moreover, semi-IPN is favorable strategies for compatibilizing immiscible polymers. The network features can be desired by polymer type, polymer concentration, and applied cross-linking method and also by the general protocol used for their preparation (14-16).

Poly(ɛ-caprolactone) and poly(lactide)s from polyesters family which are used in many biomedical applications, have gained a great attention due to their biodegradability and miscibility with a variety of polymers. In addition, poly (ester urethanes) which contain PCL, PLA blocks and diisocyanate in their structure have good elastomer properties and biocompatibility as well as high physical and mechanical properties. Currently, the only technically reasonable method for producing polyurethane (PU) plastics is the poly addition process of diisocyanates and polyols. Hence, the manufacturing methods and properties of the isocyanate feed stocks are the deciding factors for the production of polyurethanes (17-21).

As it is known from literature, there are two part starting materials in the preparation of polyurethanes, one as chain extender typically various types of diisocyanate and other, polyethers such as polyethylene oxide diols or polyester such as polycaprolactone (PCL) diols as convenient long chain diols. The aromatic diisocyanate such as 4, 4′-diisocyanatodiphenylmethane (MDI) includes toxic and carcinogenic degradation product, therefore is generally used in the industrial preparation of Pus and has to be excluded from the synthesis of biodegradable Pus. The alternative aliphatic diisocyanate such as 1,6-diisocyanatohexan (HDI) which is used in the present work is preferred, because its degradation product are nontoxic or low toxic and metabolized or eliminated by the living organism (22-24). 

As it was mentioned before, it is great of interest to make careful choice of the reactant to avoid the dangerous degradable products. There are several polyol which is nontoxic and can be used as a reactant in the preparation of Pus. Polycaprolactone is biodegradable polyester. It has a methylene group on the main chain which gives hydrophobic character to the polymer, leading long times of degradation in aqueous media. 6-hydroxyhexanoiec acid is degradation product of PCL which is transformed by microsomal ɷ-oxidation to adipic acid, a natural occurring metabolite. PCL preferred in this work due to these tremendous properties (22-27).

**Scheme 1:**
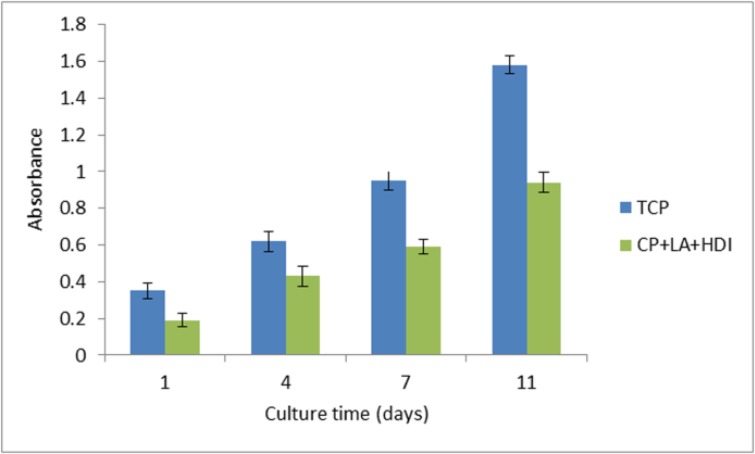
Synthetic scheme of the biodegradable polymer network

As part of our interest in the synthesis of new organic and polymeric compounds and its biomedical application, (28-31) in this present research, it has been tried to report the synthesis of a new type of semi-interpenetrating polymer networks, derived from ɛ-caprolactone, lactide, 1,4-butane diisocyanate and ethylenediamine and also its potential in the soft tissue engineering applications (Scheme 1) (32).

## Experimental

*Materials*

Lactide (LA) was obtained from Acros Company. ɛ-Caprolactone (CL) was purchased from Sigma and purified with CaH_2_ by vacuum distillation. Stannous octoate (Sn(Oct)_2_, 95%) was purchased from Aldrich and used as received. 1,4-Butane diisocyanate (Merck) and ethylenediamine (Merck) used as received. Solvent dimethylsulfoxide (DMSO) was dried with 3Å molecular sieve. All other commercially available solvents were purchased from Merck Chemical Co. and used as received. Dulbecco’s Modified Eagle’s Medium (DMEM)/F12, RPMI 1640 and fetal calf serum (FCS) were obtained from GIBCO Invitrogen Corporation. 3-(4,5 Dimethylthiazol-2-yl)-2,5-diphenyl tetrazolium bromide (MTT), dimethyl sulfoxide (DMSO) and epidermal growth factor (EGF) were purchased from Sigma. 

*CL/LA prepolymer synthesis*

The 4-arm star-Shaped CL/LA prepolymer was synthesized by Sn (Oct)_2_-catalyzed ring-opening polymerization of CL and LA. Typical polymerization procedure was as following: A certain amount of pentaerythritol (0.13 g, 1 mmol), Sn(Oct)_2_ (0.2 wt.% of monomers), LA (9.12 g, 80 mmol ) and CL (9.12 g, 80 mmol ) were placed in a three-neck round-bottom flask equipped with a reflux condenser under a nitrogen atmosphere. Then, the reaction vessel was put in oil bath at 120 ^°^C, for 24 h with stirring. After the reaction flask was cooled to room temperature, the resulting product was dissolved in methylene chloride and then poured into excess methanol to precipitate the polymerized product. The 4-arm star shaped CL/LA prepolymer with a hydroxyl group at each chain end was obtained after filtering and drying in a vacuum at room temperature for 48 h. Yield: 8.38 g (90%) 

(20, 33). 

*Polymer network synthesis*

In a second step, stoichiometry of the reaction was 4:2:2 of 1, 4-butane diisocyanate (BDI): prepolymer (PLA/PCL): ethylenediamine. An approximate 25 wt% solution of prepolymer in DMSO was mixed with an approximate 15 wt% solution of BDI in DMSO. Stannous octoate (0.2 wt%, with respect to monomers) were then added. 

This compound was allowed to react at 70 °C for 3 h. The solution was cooled to room temperature, the ethylenediamine solution was added dropwise under stirring and the reaction was continued at room temperature for a period of 24 h. The polymer solution was precipitated in distilled water. Finally, the polymer was dried in freeze dryer for 24 h (20, 34-38).

*Polymer characterization*

The chemical structure of polymer was confirmed by FT-IR and ^1^H NMR spectra. ^1^H NMR was recorded on a Bruker DRX-300 Avance spectrometer (300 MHz) with CDCl_3_ as a solvent. The molecular weight of the resulting polymer was also determined by ^1^H NMR. The thermal transitions of the polymers were observed with a differential scanning calorimeter (DSC) at a heating rate of 20 K/min over the temperature range of 0 to 800 ºC. Phase transitions and enthalpy were studied by differential thermal analysis (DTA) and finally, thermal gravimetric analysis (TGA) was used to evaluate the thermal stability of a polymer over the temperature range of 0 to 800 ºC (39, 19, 40, 21, 41 and 42).

*Hydrolytic degradation tests*

Polymer degradation in phosphate buffered saline (PBS) at 37 ºC was characterized over 8 week period. To measure polymer hydrolytic degradation, polymer was weighed and immersed in 10 mL PBS (pH = 7.4) at 37 ºC. Samples were taken every week and weighed after washing with fresh PBS and then drying in a freeze dryer for 4 days. The weight remaining was calculated as weight remaining (%) = 100 × W2/W1 

Where W1 and W2 are the weights of polymer before and after degradation, respectively 

(43-48).

*Biocompatibility test of polymer*

Human Skin Fibroblast Foreskin of a male newborn (HNFF-PI8) cells were grown in 50-mL cell-culture flasks with Dulbecco’s modified Eagle’s medium (DMEM, Gibco) supplemented with 10% fetal bovine serum (Gibco), 0.3 mg/mL of Lglutamine (Gibco), 0.05 mg/mL of ascorbic acid, 3.7 mg/mL of NaHCO_3_ (Sigma), and 100 Units/mL each of penicillin and streptomycin. After obtaining adequate confluence, the cells were detached by trypsin and counted. Then the cells were seeded onto tissue culture plate (TCP) as control and polymer films, placed in a 24-well plate with the density of 1×108 cell per well and cultured with Dulbecco›s modification of Eagle›s medium (DMEM) containing 15% fetal calf serum (FCS) supplement (19, 40 and 49). After cell culturing for 1 to 11 days, the viability and proliferation of fibroblast cells was determined by MTT (3-[4, 5-dimethylthiazol-2-yl]-2,5 diphenyltetrazolium bromide) assay. Firstly, 5 mL of MTT solution (5 mg/mL) were added to the culture wells and they were incubated at 37 °C and 5% CO_2_ for 4 h. The upper medium was removed cautiously and the intracellular Formosan was solubilized by adding 2 mL DMSO to each well. The absorbance of produced Formosan was measured at 570 nm with a spectrophotometer (Convergys) (21).

## Results and Discussion

*Ring-opening polymerization of CL/LA*

Polymer was synthesized by the ring-opening polymerization of CL and LA with pentaerythritol as an initiator and Sn (Oct)_2_ as a catalyst. The [CL]/[LA] molar ratio was 1/1, and 1,4-butane diisocyanate (BDI) with ethylenediamine used as a chain cross linker. BDI was chosen as the diisocyanate upon which the hard segment was constructed since it would be assumed to yield ethylenediamine, a polyamine that is necessary for cell growth and differentiation, following complete degradation (50). Figure 2(a) shows the typical ^1^H NMR spectrum of CL/LA prepolymer. Calculations based on the ^1^H NMR spectrums represent the molecular weight of 8578 g/mol for the prepolymer. 

*Synthesis and characterization of CA/LA prepolymer and PU polymer*

**Figure 1:**
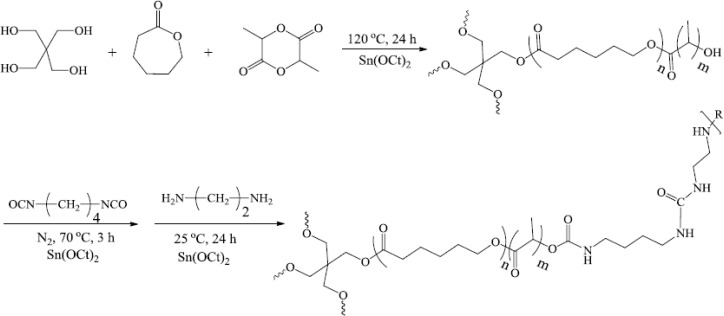
Typical ^1^H NMR spectrum of (a) CL–LA prepolymer and (b) polymer network

^1^H NMR characterized the composition and relative component molecular weights for C-[P(CL-*b*-LA)]_4_ and C-[P(CL-*b*-LA)-N=C=O]_4_. Measurements were made at room temperature with chloroform as a solvent and a polymer concentration of approximately 15 mg/mL. Figure 1(b) shows the ^1^H NMR spectra of the polymer network. The H signals of repeating methylene units adjacent to a carbonyl group in PCL block appear in *δ* = 2.28 ppm, OCOC*H*_2_ (b), *δ* = 4.05 ppm, C*H*_2_O-CO (f), also in PLA block: *δ* = 5.16 ppm related to CO-C*H*(Me)OH (g) and OH group in *δ* = 4.86 ppm as a broad signal were quite distinguishable for the ^1^H NMR spectra of prepolymer. 

The integral ratio of these proton signals were found to be in good agreement with the proposed structure of prepolymer. As showed in Figure 1(b), the ^1^H NMR of PU network in comparison to prepolymer (Figure 1(a)) confirmed the new appeared signals in spectra as follow: *δ* = 3.40 ppm, C*H*_2_-NH and C*H*_2_-NH_2_ (j), *δ* = 2.32 ppm for C*H*_2_-CH_2_NHCO (k), a broad signal at *δ* = 4.36 ppm for NH_2_ terminal (l).

**Figure 2:**
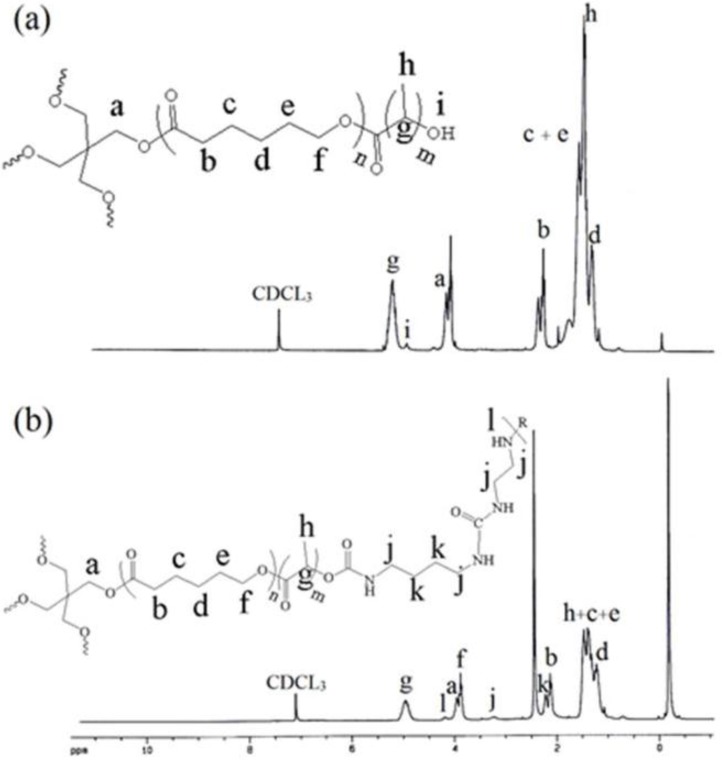
Infrared spectra of the (A) CL/LA prepolymer and (B) polymer network

Figure 2. shows the FT-IR spectra of the prepolymer (a) and polymer network (b). The broad absorption band of OH stretching vibrations for prepolymer at 3340 cm^-1^ disappeared for polymer. New absorption bands of the NH and NH_2_ stretching vibrations and NH bending vibrations appeared at 3400 and 1460 cm^-1^ for polymer, respectively. These results suggest that the reaction of the hydroxyl group of prepolymer and the isocyanate group of BDI proceeded smoothly to generate the polymer network by urethane linkages.

**Table1:** Thermal properties of CA/LA prepolymer and PU polymer

Samples	T_m_ Ċ	T_g_ (Ċ)
CA/LA prepolymer	**339.5**	**58.1**
PU polymer	**326.9**	**151.6**

**Figure 3:**
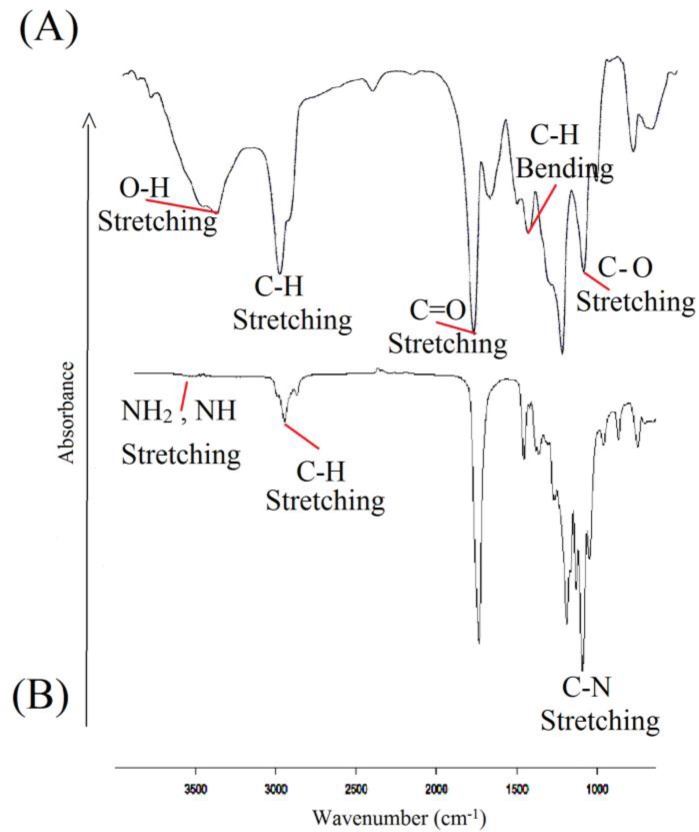
TGA analysis for (A) CL/LA prepolymer and (B) PU polymer

**Figure 4:**
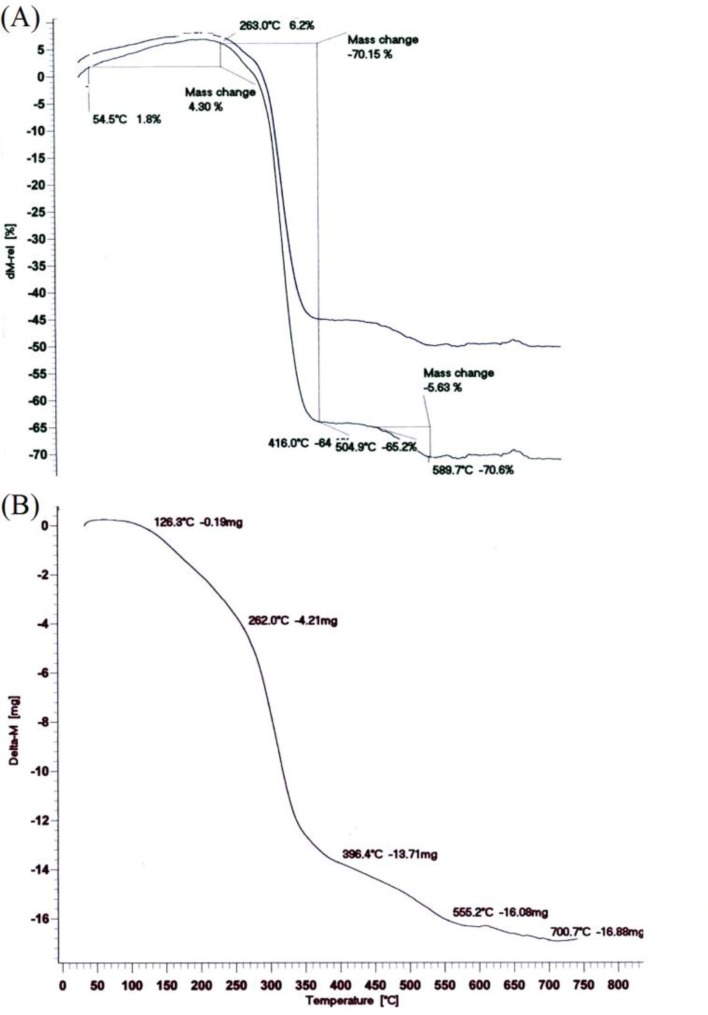
Thermal analysis of DSC for (A) CL/LA prepolymer, (B) PU polymer and DTA for (C) CL/LA prepolymer, (D) PU polymer

**Figure 5:**
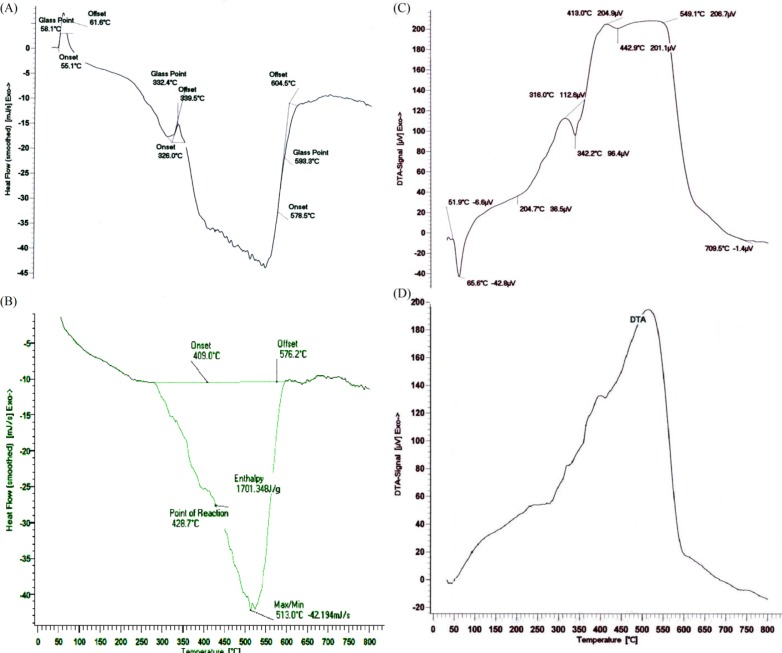
Weight loss of PU network during hydrolytic degradation at 37 ºC

**Figure 6:**
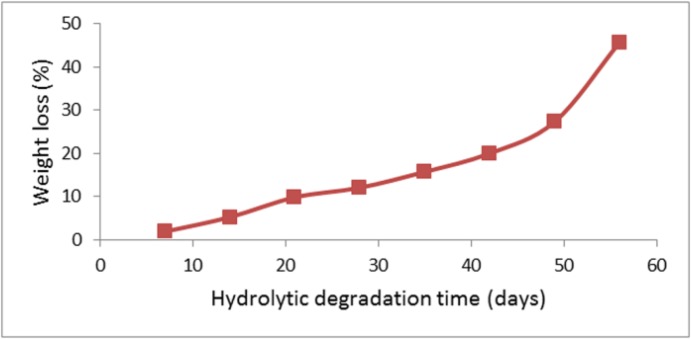
FT-IR spectra of PU network degradation, (a) PU powder, (b) after 2 weeks and (c) after 6 weeks

**Figure 7:**
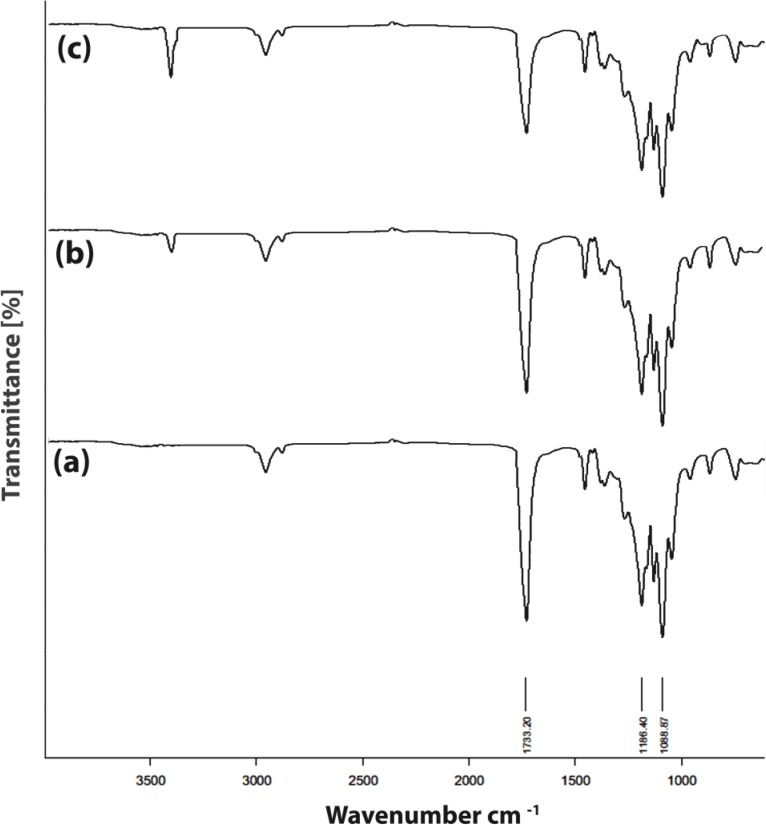
Cell adhesion assay of HNFF-PI8 cells on PU polymer. Absorbance at 570 nm is represented for progressive culture times. Culture plastic was used as a control. Data are expressed as means of a representative of three similar experiments carried out in triplicate

Temperature changes in the TGA analysis make changes in the polymer mass. In fact, the results are restricted to the decomposition reactions, oxidation and physical processes such as evaporation. According to the Figure 3(a). a volume increase is observed in the range of 50-260 ºC, which represents an oxidation. The diagram shows three stages of degradation. In the first stage, the LA degradation is observed up to 416 ºC and in the second one; there main LA and the oxides which were formed on initial temperatures are destroyed in the range of 416-589 ºC. Also, the CP degradation finally occurred above 589 ºC. As TGA spectrum of the polymer shows in Figure 3(b), there are three steps of weight lose. The weight loss in the timeframe of 100-150 ^°^C was due to desorption of water molecules from the surface which was estimated that to be about 20%. In the timeframe of 180-370 ^◦^C, weight loss took place in different rate that was attributed to the degradation and decomposition of the polymer (51, 41, 42, 36, 52, 38 and 20).

The thermal parameters of the synthesized PU network were determined by DSC with typical curves shown in Figure 4 (a and b). The glass transition temperature, Tg, was considered as the point of the DSC scan inflection. At this temperature, as a result of starting a matched movement of large segments of the polymer molecules, the disfigured polymer compositions become rubbery and elastic. Based on the initial curves, enthalpy will not change with heating to a certain temperature, because the plastic deformation process does not involve heat absorption or diffusion, but due to changes in heat capacity, the base line will change. Thus, according to the enthalpy changes which are equal to zero, peaks will not be achieved during this transition.

The peak temperature, which is a sign for the material identification, in the Figure 4(c) is about 425 ºC. The peak shape indicates the type of thermal event which has appeared. Changes in crystal structure and melting emerge as a sharp peak which is around 330 ºC for prepolymer. The thermal decomposition and chemical reactions occur in a broad peak. As is clear from Figure 4(d), the polymer is decomposed in the temperature range 400-600 ºC. According to the spectrum obtained for the PU polymer, the peak temperature is about 520 ºC. Polymer begins to melt at 326 ºC. Also in the temperature range from 360 to 620 ºC, we have the phenomenon of polymer degradation.

The results of thermal analyzes indicate the melting point and the glass temperature of the CA/LA prepolymer and PU polymer which are given in the Table 1.

*Hydrolytic degradation of the PU*

The hydrolytic degradation rates of PU network were determined by measuring its weight loss during its hydrolytic degradation. Figure 5. shows the hydrolytic degradation of PU network at 37 ºC. The polymer exhibited progressive mass loss over the 8 week period ranging from 1.90% to 45.66%. The chemical structure of PU network was confirmed by FT-IR spectra recorded. It was said that the hydrolytic degradation rate of aliphatic polyesters relies on the morphological structure, crystallinity, size and form of the crystallite, *etc*. (43). Since water molecules can diffuse into the amorphous area of the polymer without difficulty, the hydrolytic degradation happens in the amorphous area rather than the crystalline area. It was found that the presence of LA units of PU network reduced its crystallinity and resulted in the improvement of its hydrolytic degradation.

FT-IR spectra for the degradation of the polymer after 2 and 6 weeks in PBS are shown in Figure 6. The peak at 1733 cm^-1^ corresponds to ester and urethane stretching vibrations (44). The results of FT-IR analysis show the reduction in the peaks intensity which is caused by polymer destruction. As is clear, a sharp decline in the intensity of peaks, especially the peak of the ester groups, can be seen after six weeks. This indicates the flexibility of polymer degradation with time. This observation implies that the PU network degrades by hydrolysis of ester linkages to yield hydroxyl acids. 

*Biocompatibility of polymer*

Cell proliferation on the PU polymer and tissue culture plate (TCP) was measured by MTT solution (5 mg/mL). The MTT assay is established on the reduction of the yellow tetrazolium salt to purple formazen crystals by dehydrogenase enzymes emitted from the mitochondria of metabolically active cells. Figure 7. shows the viability graph of the cells cultured on the polymer and tissue Culture plate (TCP) as control. After 1, 4, 7 and 11 days of cell seeding in 24-well dish, the original medium was removed and 100 μL fresh medium and 10 μL MTT solution were added to each well. After 4-hour incubation at 37 ºC in 5% CO_2_, MTT solution from each well was carefully removed and replaced by 40 μL DMSO for each well. Then the absorbance of solution was measured at 570 nm.

## Conclusion

The polyurethane (PU) polymer was synthesized from ɛ -caprolactone and lactide with using a ring opening polymerization and characterized by different analysis such as ^1^H NMR and FT-IR. The degradation rate of polymer was evaluated at a controlled condition and the results showed that the decomposition products were non-cytotoxic. The PU polymer also supports the attachment and proliferation of HNFF-PI8 cells. According to these suitable properties, the PU polymer has a great potential as biodegradable scaffolds for soft tissue engineering applications.

## References

[1] Ting-Yu Shih J-DY, Hsi-Wei Jia, Jui-Hsiang Chen (2013). Synthesis and Properties of Novel Biodegradable Segmented Poly (ε-caprolactone). J Med Biol Eng..

[2] Florian K (2010). Wolf AMFaHF Poly(glycolide) multi-arm star polymers: Improved solubility via limited arm length. Beilstein J Org Chem.

[3] J Z Du LYT, W J Song, Y Shi (2009). Evaluation of polymeric micelles from brush polymer with poly(epsilon-caprolactone)-b-poly (ethylene glycol) side chains as drug carrier,”. Biomacromolecules..

[4] Astrid Hoppe FS, Claire-Hélène Brachais, Gilles Boni, Jean-Pierre, Couvercelle, Laurent Plasseraud (2013). 1-n-Butyl-3-methylimidazolium-2-carboxylate: a versatile precatalyst for the ring-opening polymerization of ε-caprolactone and rac-lactide under solvent-free conditions. Beilstein J Org Chem..

[5] Hadjichristidis N, Pispas S, Floudas G (2003). Block copolymers: synthetic strategies, physical properties, and applications. Wiley. Com..

[6] Bellas V, Rehahn M (2007). Universal methodology for block copolymer synthesis. Macromolecular rapid commun...

[7] Buwalda SJ1 DP, Calucci L, Forte C, Feijen J (2010). Influence of amide versus ester linkages on the properties of eight-armed PEG-PLA star block copolymer hydrogels. Biomacromolecules..

[8] Armin Burgath AS, Ingo Neuner, Rolf Mülhaupt, Holger Frey (2000). Multi-arm star block copolymers based on ε-caprolactone with hyperbranched polyglycerol core. Macromolecular Chem. Phys..

[9] Lapienis G (2009). Star-shaped polymers having PEO arms. Prog. Polym. Science.

[10] Eschwey H, Hallensleben ML, Burchard W (1973). Preparation and some properties of star-shaped polymers with more than hundred side chains. Die Macromolecular Chemie..

[11] Fujimoto T, Tani S, Takano K, Ogawa M, Nagasawa M (1978). Preparation and characterization of a starshaped polymer. Macromolecules..

[12] Matricardi P, Di Meo C, Coviello T, Hennink WE, Alhaique F (2013). Interpenetrating Polymer Networks polysaccharide hydrogels for drug delivery and tissue engineering. Advanced Drug Delivery Reviews..

[13] AD Jenkins PK, RFT Stepto, UW Suter (1996). Glossary of basic terms in polymer science (IUPAC Recommendations 1996). Pure Appl Chem..

[14] Shibita A, Takase H, Shibata M (2014). Semi-interpenetrating polymer networks composed of poly(l-lactide) and diisocyanate-bridged 4-arm star-shaped ɛ-caprolactone oligomers. Polymer.

[15] Pan H, Pu H, Jin M, Wan D, Modestov AD (2013). Semi-interpenetrating polymer networks based-on endgroup crosslinked fluorine-containing polyimide via click chemistry. Electrochimica Acta..

[16] Merlin DL, Sivasankar B (2009). Synthesis and characterization of semi-interpenetrating polymer networks using biocompatible polyurethane and acrylamide monomer. European Polymer Journal.

[17] Lazdinaa B, Stirna U, Tupureina V, Dzene A, Sevastyanovaa I (2006). Synthesis and properties of cross-linked poly(ester urethanes) from poly (lactide) triols and poly (caprolactone) diols. Proceedings of the Estonian Academy of Sciences Chemistry.

[18] Kister G, Cassanas G, Bergounhon M, Hoarau D, Vert M (2000). Structural characterization and hydrolytic degradation of solid copolymers of d, l-lactide-co-ε-caprolactone by Raman spectroscopy. Polymer.

[19] Gautam S, Dinda AK, Mishra NC (2013). Fabrication and characterization of PCL/gelatin composite nanofibrous scaffold for tissue engineering applications by electrospinning method. Materials Science and Engineering: C..

[20] Peponi L, Navarro-Baena I, Sonseca A, Gimenez E, Marcos-Fernandez A, Kenny JM (2013). Synthesis and characterization of PCL-PLLA polyurethane with shape memory behavior. European Polymer Journal..

[21] Yang J, Shi G, Bei J, Wang S, Cao Y, Shang Q, Yang G, Wang W (2002). Fabrication and surface modification of macroporous poly (L-lactic acid) and poly (L-lactic-co-glycolic acid)(70/30) cell scaffolds for human skin fibroblast cell culture. Journal of biomedical materials research..

[22] Marcos-Ferna´ndez A, Abraham G A, Valentı´n J L, San Roma´n J (2006). Synthesis and characterization of biodegradable non-toxic poly(ester-urethane-urea)s based on poly(3-caprolactone) and amino acid derivatives. Polymer.

[23] Jian H, Bing C, Lin Y, Ai-ying Z, Jian Z, Zeng-guo F (2009). Synthesis and characterization of biodegradable polyurethane based on poly(ε-caprolactone) and L-lysine ethyl ester diisocyanate. Frontiers of Materials Science China.

[24] José E Báez, Daniel R, Juan L V, Angel M-F (2012). Biodegradable Poly(ester−urethane−amide)s Based on Poly(ε- caprolactone) and Diamide−Diol Chain Extenders with Crystalline Hard Segments. Synthesis and Characterization. Macromolecules,.

[25] Siyanbola T O, Sasidhar K, Anjaneyulu B, Kumar K P, Rao B V S K, Ramanuj N, Olaofe O, Akintayo E T, Raju K V S N (2013). Anti-microbial and anti-corrosive poly (ester amide urethane) siloxane modified ZnO hybrid coatings from Thevetia peruviana seed oil. Journal of Material Science.

[26] Jukka T, Janne K, Anu K, Olli V, Merja I, Jukka S (2002). Biodegradation of Lactic Acid Based Polymers under Controlled Composting Conditions and Evaluation of the Ecotoxicological Impact. Biomacromolecules.

[27] Lise M, Thomas L, Thi H N N, Benoit G, Carine A, Henri C (2012). Hydroxyl telechelic building blocks from fatty acid methyl esters for the synthesis of poly(ester/amide urethane)s with versatile properties. Polymer Chemistry.

[28] Rezayan AH, Azerang P, Sardari S, Sarvary A (2012). Synthesis and biological evaluation of coumarin derivatives as inhibitors of Mycobacterium bovis (BCG). Chemical biology & drug design.

[29] Shaabani A, Rezayan AH, Keshipour S, Sarvary A, Ng SW (2009). A novel one-pot three-(in situ five) component condensation reaction: an unexpected approach for the synthesis of tetrahydro-2, 4-dioxo-1 Hbenzo[b][1, 5] diazepine-3-yl-2-methylpropanamide derivatives. Organic letters..

[30] Tabatabaei Rezaei SJ, Nabid MR, Niknejad H, Entezami AA (2012). Multifunctional and thermoresponsive unimolecular micelles for tumor-targeted delivery and site-specifically release of anticancer drugs. Polymer..

[31] Nabid MR, Tabatabaei Rezaei SJ, Sedghi R, Niknejad H, Entezami AA, Oskooie HA, Heravi MM (2011). Selfassembled micelles of well-defined pentaerythritol-centered amphiphilic A< sub> 4</sub> B< sub> 8</sub> starblock copolymers based on PCL and PEG for hydrophobic drug delivery. Polymer..

[32] Gunatillake PA (2003). Adhikari R Biodegradable synthetic polymers for tissue engineering. Eur Cell Mater..

[33] Krol P (2007). Synthesis methods, chemical structures and phase structures of linear polyurethanes.Properties and applications of linear polyurethanes in polyurethane elastomers, copolymers and ionomers. Progress in materials science..

[34] Efstathiou K Synthesis and characterization of a Polyurethane Prepolymer for the development of a novel Acrylate-based polymer foam.

[35] Guan J, Sacks MS, Beckman EJ, Wagner WR (2002). Synthesis, characterization, and cytocompatibility of elastomeric, biodegradable poly (ester-urethane) ureas based on poly (caprolactone) and putrescine. Journal of biomedical materials research..

[36] Guan J, Sacks MS, Beckman EJ, Wagner WR (2004). Biodegradable poly (ether ester urethane) urea elastomers based on poly (ether ester) triblock copolymers and putrescine: synthesis, characterization and cytocompatibility. Biomaterials..

[37] Joshi VP (2009). Studies on synthesis & characterization of thermoplastic polyurethane-urea copolymers. Dissertation.

[38] Zhang C, Wen X, Vyavahare NR, Boland T (2008). Synthesis and characterization of biodegradable elastomeric polyurethane scaffolds fabricated by the inkjet technique. Biomaterials..

[39] Mythili C, Retna AM, Gopalakrishnan S (2004). Synthesis, mechanical, thermal and chemical properties of polyurethanes based on cardanol. Bulletin of Materials Science..

[40] Park C-H, Kim EK, Tijing LD, Amarjargal A, Pant HR, Kim CS, Shon HK (2014). Preparation and characterization of LA/PCL composite fibers containing beta tricalcium phosphate (β-TCP) particles. Ceramics International..

[41] Zhang X, Xu R, Wu Z, Zhou C (2003). The synthesis and characterization of polyurethane/clay nanocomposites. Polymer international..

[42] Cervantes-Uc J, Espinosa J, Cauich-Rodríguez J, Ávila-Ortega A, Vázquez-Torres H, Marcos-Fernández A, San Román J (2009). TGA/FTIR studies of segmented aliphatic polyurethanes and their nanocomposites prepared with commercial montmorillonites. Polymer Degradation and Stability..

[43] You Y, Min BM, Lee SJ, Lee TS, Park WH (2005). In-vitro degradation behavior of electrospun polyglycolide, polylactide, and poly (lactide-co-glycolide). Journal of Applied Polymer Science..

[44] Guelcher SA, Srinivasan A, Dumas JE, Didier JE, McBride S, Hollinger JO (2008). Synthesis, mechanical properties, biocompatibility, and biodegradation of polyurethane networks from lysine polyisocyanates. Biomaterials..

[45] Lipika VT, Konga JF, Chattopadhyaya S, Widjajaa LK, Liowa SS, Venkatramana SS, Marc JM Abadiea (2010). Thermoplastic biodegradable elastomers based on e-caprolactone andL-lactide block co-polymers: A new synthetic approach. Acta Biomaterialia..

[46] Grad S, Kupcsik L, Gorna K, Gogolewski S, Alini M (2003). The use of biodegradable polyurethane scaffolds for cartilage tissue engineering: potential and limitations. Biomaterials..

[47] Middleton JC, Tipton AJ (2000). Synthetic biodegradable polymers as orthopedic devices. Biomaterials..

[48] Patel H, Bonde M, Srinivasan G (2011). Biodegradable Polymer Scaffold for Tissue Engineering. Trends in Biomaterials and Artificial Organs..

[49] MC Serranoa, RP, M Vallet-Reg! ıb, J Pen, A R! amilab, I Izquierdob, MT. Portol (2004). In-vitro biocompatibility assessment of poly(e-caprolactone) films usingL929 mouse fibroblasts. Biomaterials.

[50] Guan J, Fujimoto KL, Sacks MS, Wagner WR (2005). Preparation and characterization of highly porous, biodegradable polyurethane scaffolds for soft tissue applications. Biomaterials..

[51] Trovati G, Sanches EA, Neto SC, Mascarenhas YP, Chierice GO (2010). Characterization of polyurethane resins by FTIR, TGA, and XRD. Journal of Applied Polymer Science..

[52] Lee DC, Jang LW (1996). Preparation and characterization of PMMA–clay hybrid composite by emulsion polymerization. Journal of Applied Polymer Science..

